# Assessment of the Effect of Oral Health on Quality of Life and Oral-Health Indicators among ESRD Patients in Southwest Florida: A Pilot Study

**DOI:** 10.1155/2019/1608329

**Published:** 2019-09-23

**Authors:** Payal Kahar, Carol Chapman, Jayanta Gupta

**Affiliations:** ^1^Department of Health Sciences, Florida Gulf Coast University, Fort Myers, Florida, USA; ^2^Department of Dental Hygiene, Florida Southwestern College, Fort Myers, Florida, USA

## Abstract

**Purpose:**

To determine and compare OHRQoL (oral-health-related quality of life) using the Geriatric Oral Health Assessment Index (GOHAI-12) and Oral Health Impact Profile (OHIP-14) among patients receiving hemodialysis (HD).

**Methods:**

Face-to-face interviews and intraoral examinations were conducted among 70 patients. Mann–Whitney *U* test and Kruskal–Wallis test were used to compare each item score with demographics and dental and overall health status.

**Results:**

The mean number of years on dialysis was 4.7 ± 7.5 yrs; the mean number of teeth present was 19.7 ± 11.04; median values of OHRQoL using GOHAI-12 and OHIP-14 were 52 and 64. Within GOHAI-12, limiting food (*p* 0.043), uncomfortable eating in front of people (*p* 0.045), limiting contact with people (*p* 0.046), and eating without discomfort (*p* 0.011) were significantly associated with females. Being worried (*p* 0.040) and self-conscious (*p* 0.048) were significant for age groups ≤65 years. Prevented from speaking was associated with >20 teeth (*p* 0.016). Being worried about oral health was associated with number of years on dialysis (*p* 0.042). Within OHIP-14, speech was associated with number of teeth present (*p* 0.024). Total inability to function was significantly associated with race (*p* 0.018), number of teeth (*p* 0.028), and edentulousness (*p* 0.031).

**Conclusions:**

GOHAI-12 was more effective than OHIP-14 in assessing OHRQoL. However, most subjective experiences did not correlate with clinical findings. Systemic health issue like end-stage renal disease affecting QoL might have taken precedence over dental problems. Clinical assessments should be inherent in oral-health evaluation and there should be cooperation between nephrologists and dentists in promoting oral health and treating systemic conditions among HD patients.

## 1. Introduction

The World Health Organization definition and successive redefining of health emphasize that there are different and related forms of wellness and that the absence of functional disabilities most aptly defines health [1–3]. People view health and illness to exist along a continuum, where wellness and illness represent subjective experiences [2]. With the redefining of health, there has been an increased emphasis on assessing subjective experiences of wellness and illness not only among people across different sociodemographic and settings but among those with chronic diseases such as end-stage renal disease patients (ESRD) [4, 5].

The number of ESRD prevalent cases continues to rise by 21,000 cases per year in the United States [6]. Not only do ESRD patients experience a myriad of oral-health-related problems such as periodontitis, xerostomia, changes in teeth, and taste linked to their existing systemic conditions and medications, but also existing oral-health problems could worsen their existing comorbidities [7–10]. While clinical oral examinations have been used traditionally to evaluate oral health, oral health defined in terms of person-centered, subjective health experiences help understand oral-health-related quality of life (OHRQoL).

OHRQoL questionnaires developed so far measure the outcomes of oral and orofacial disorders in general [11]. They quantify “burden of oral diseases” by shifting focus from traditional dental assessments that rely on objective measures of presence or absence of oral diseases to a person's physical functioning and social and emotional experience in defining oral-health outcomes [12]. OHRQoL correlates with psychological assets such as optimism and resilience [13]. Various instruments measure OHRQoL of which Geriatric (General) Oral Health Assessment Index (GOHAI-12) and Oral Health Impact Profile (OHIP-14) have been widely used in studies of both younger and older populations [11, 14–16].

GOHAI-12 and OHIP-14 possess several differences between the two; however, many OHRQoL measures in the two instruments appear to be quite similar as well. [11, 14–16] Studies using GOHAI-12 and OHIP-14 have focused mainly on healthy and older individuals across different sociodemographics [14–16]. Further research comparing the performance of different measures with respect to their ability to detect OHRQoL in different populations and settings is needed. A few studies that presented data on ORHQoL among ESRD patients in Poland, Germany, and Iran had no consistent findings [17–20]. Subjective measures of oral-health outcomes correlated with clinical findings in only two studies but did not in the other studies [18, 19]. The ability of the dimensions in GOHAI-12 and OHIP-14 to predict and measure OHRQoL also differed in the studies [17–20]. Furthermore, none of the studies were done in the United States.

Hence, the purpose of the study was to determine OHRQoL using GOHAI-12 and OHIP-14 and oral-health status among ESRD patients in southwest Florida. Secondly, the purpose was to compare GOHAI-12 and OHIP-14 in describing OHRQoL.

## 2. Methods

### 2.1. Research Design and Procedure

This cross-sectional study was conducted at three dialysis centers in southwest Florida during October 2017 through July 2018. Two trained research assistants and a dental hygienist conducted the interviews and clinical examinations, respectively. Interviews were conducted face-to-face using the GOHAI-12 and OHIP-14 questionnaires while patients were receiving hemodialysis. Intraoral examinations were carried out using dental headlights under room lights by chairside at the dialysis centers. The institutional review board committee at two Florida universities, a kidney care provider, and the three dialysis centers approved the study.

### 2.2. Participants

Participation in the study was both anonymous and voluntary. The inclusion criteria were as follows: adults over the age of 18 years receiving hemodialysis who were either edentulous or dentulous. Individuals who did not sign the written consent and were unable to comprehend the questions due to language barriers were excluded from the study. Approximately 120 patients were approached, out of which 70 were enrolled in the study after applying the exclusion criteria.

### 2.3. Measures

#### 2.3.1. Demographics

Information was collected on age, gender, and race. Age was treated as a categorical variable and subdivided into two groups: ≤65 years and >65 years. One of the most significant demographic trends in the US is the increased proportion of the age group 65 years and older. The state of Florida has a higher percentage (20.5) of elderly above the age of 65 years than the national average (16) [21]. While assessment of full effects of dental decay, extent of periodontal involvement and effects of dental care provided is possible in both age groups, estimation of oral diseases from a life course perspective is more conceivable with age group older than 65 years [22].

#### 2.3.2. Nonclinical Measures

For assessments of OHRQoL, previously validated GOHAI-12 and OHIP-14 questionnaires were used [16, 23, 24]. For the GOHAI-12 questionnaire, participants had to indicate how often they experienced an oral-health problem in the last three months in a 6-point Likert scale, the frequency of which varied from never, seldom, sometimes, often, and very often to always [16]. All, but three questions that were positively directed out of the twelve questions, were given a value of 5 for never and 0 for always. The sum ranged from 0 to 60, with higher scores corresponding to better oral health. For OHIP-14, fourteen questions elicited frequency of oral-health problems experienced in the last year ranging from don't know, never, hardly ever, occasionally, and fairly often to very often [25]. Never was given a value of 5, very often a value of 1, and don't know was given a value of 0. The sum score could range from 0–70; the higher the score, the fewer were the oral-health-related problems. Cronbach's alpha for GOHAI-12 and OHIP-14 were 0.91 and 0.92, respectively, indicating high internal consistency.

#### 2.3.3. Clinical Measures

A trained dental hygienist carried out the dental examinations using dental mirror and periodontal probe. Decayed, missing, and filled teeth were recorded, and the number of remaining teeth was calculated from the missing teeth index. Dental decay was recorded as present if a lesion in a pit or fissure or a smooth surface on a tooth had a definite cavity, undermined enamel or a noticeably softened floor or wall [22]. The remaining number of teeth was treated as a categorical variable and divided into two groups: <20 teeth and ≥20. A count of 20 teeth or a certain number of occluding posterior teeth is defined as satisfactory oral health [26–28]. Community Periodontal Index (CPI) ranging from 0–4 (0 = healthy; 1 = bleeding on probing; 2 = calculus; 3 = pocket 4-5 mm; 4 = pocket 6 mm or more) was determined in the six sextants of the oral cavity for each participant except in cases where there were no index teeth to examine [22]. Overall CPI was the highest value noted in all the six sextants. Diabetes status and years on dialysis were determined through self-reports. Saliva PH ranging from 5–7.8 was assessed using a saliva-testing examination tool.

#### 2.3.4. Data Analyses

Complete data were entered into a SPSS database (IBM Corp., version 24, Armonk, NY). Descriptive statistics are presented as percentages of participants responding always, very often, often, and sometimes to each GOHAI-12 and OHIP-14 item. The median and the interquartile range (IQR) for each item in the two questionnaires with respect to demographic and clinical characteristics are also presented. Since the variables were not normally distributed, Mann–Whitney *U* test and Kruskal–Wallis test were used to compare each item score with two or more groups in demographic and clinical categories.

## 3. Results

**Figure 1:**
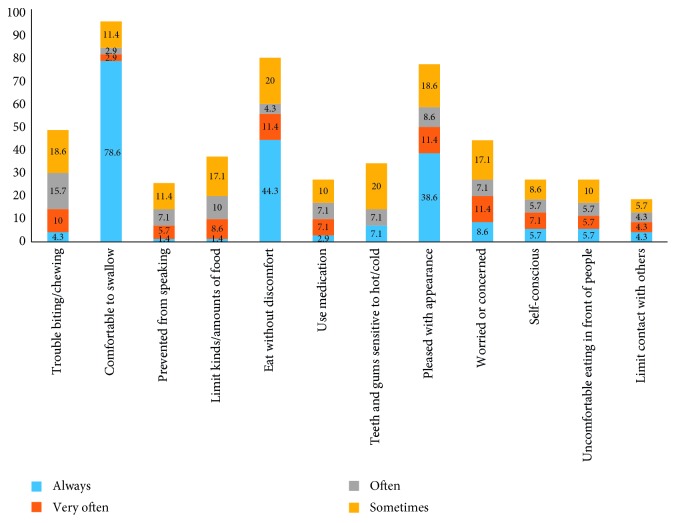
Percentage of participants responding always, very often, often, and sometimes to individual GOHAI-12 items (*N* = 70)

Figure 1 shows percentage of participants who responded always, very often, often, and sometimes to each GOHAI-12 item. Within the physical dimension for GOHAI-12, 95.8% reported they were able to swallow comfortably. Thirty-seven percent of the participants reported trouble biting/chewing foods such as meats or apples and limiting kinds or amounts of food. Eating without discomfort was reported by 80% of participants. Within psychosocial dimension, 77.2% were happy with their appearances and 44% were concerned. Teeth and gums were sensitive to hot/cold for 34.2% of the participants.

**Figure 2:**
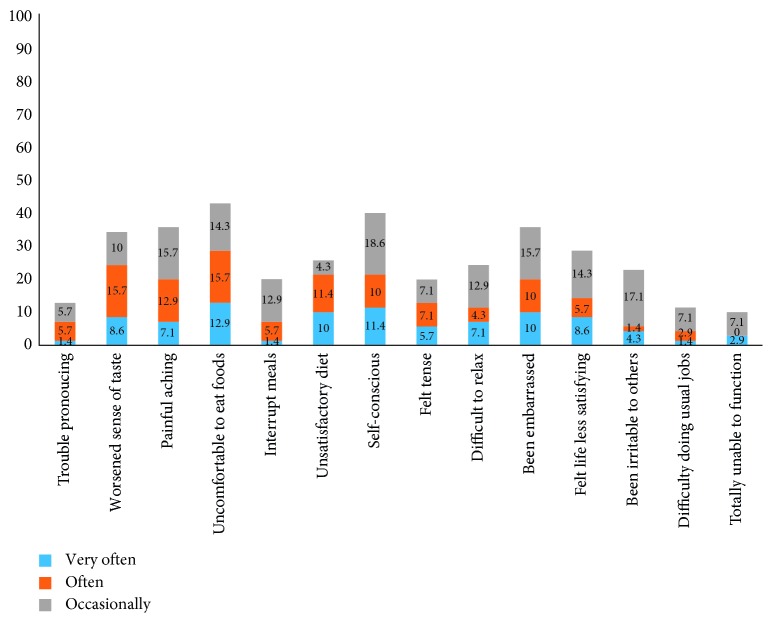
Percentage of participants responding very often, often, and occasionally to individual OHIP-14 items (*N* = 70).

Figure 2 shows the percentage of participants responding very often, often, and occasionally to each OHIP-14 item. Sense of taste worsened for 34.3% of the participants. About 43% of the participants felt discomfort while eating while 35.7% reported painful aching in the last year. About 40% reported being self-conscious and 35.7% embarrassed.

Table 1 presents the association between demographic characteristics, clinical findings, and individual GOHAI items. The median value for GOHAI-12 was 52, and the IQR varied from 39.8 to 56.3. Scores with respect to limiting food (*p* 0.043), uncomfortable eating in front of people (*p* 0.045), limiting contact with people (*p* 0.046), and eating without feeling discomfort (*p* 0.011) were significantly associated with gender, with females having lower values than males. Item scores for being worried (*p* 0.040) and being self-conscious (*p* 0.048) were statistically significant for age groups with younger patients ≤65 years having lower values than older patients >65 years. Prevented from speaking was significantly associated with the number of teeth present (*p* 0.016). Finally, being worried was significantly associated with the number of years on dialysis (*p* 0.042).

Table 2 presents the association between demographic characteristics, clinical findings, and individual OHIP items. The median value for OHIP-14 was 64 with IQR of 54.8 to 68.

Item scores for speech were significantly associated with the number of teeth present (*p* 0.024). Total inability to function was significantly associated with race (*p* 0.018), number of teeth (*p* 0.028), and edentulousness (*p* 0.031).

Table 3 shows the mean and standard deviations of decayed, missing, filled teeth index, overall CPI, and salivary PH across demographic and clinical characteristics of the study participants. Mean missing teeth index was higher among males (12.0 ± 11.1), Whites (12.1 ± 10.0), older adults <65 years (12.6 ± 10.3), and patients who reported diabetes (12.4 ± 11.2) and on dialysis for ≥3 years (12.8 ± 11.1). White participants had higher mean decayed (2.2 ± 3.8) and filled teeth index (4.8 ± 5.3). Overall mean values for CPI and saliva PH were 1.9 (1.0) and 6.9 (1.0), respectively. More number of males (*n* = 9, 22%) were edentulous than females (*n* = 3, 11.5%). More females (*n* = 11, 42.3%) had fewer than 20 teeth than males (*n* = 15, 36.5%).

## 4. Discussion

Both GOHAI-12 and OHIP-14 elicited problems with chewing or eating food, pain/sensitivity, being worried, self-consciousness, and embarrassment reflecting fewer numbers of remaining teeth, carious teeth, and gingivitis. HD patients with an overall mean age of 65 years had an average of 11 missing teeth, 2 teeth with cavities, and CPI score of 2, indicating compromised masticatory efficiency, pain, and sensitivity.

The 12 items in GOHAI-12 showed perceptions about oral health varied by age, years on dialysis, gender, and number of remaining teeth (Table 1).

Perceptions of oral health change as people age [29]. Despite having greater accumulated oral-health problems in age group >65 years, patients ≤65 years were more worried and self-conscious. Older adults with fewer teeth may have lower expectations with functionality and therefore worry less; they adapt well to their aging changes and incremental dental impairments [30–32]. Study participants reported limiting their choices of food that were easy to chew and studies show masticatory factors such as “easy to chew” food and presence of occluding pair of teeth to be associated with high OHRQoL among older people [30–32]. Patients >3 years on dialysis reported being worried, given their existing comorbid conditions that contributed to decreased QoL [33].

Females reported significantly lower scores on two psychosocial components: uncomfortable eating in front of people and limiting contact, and on one pain component: eating without discomfort in the last three months. While females in general take better care of their oral health, social appraisal is important that influences their social interactions and worries regarding social contact [34]. A previous study showed females to have lower GOHAI scores, more complaints about their discomfort, and higher expectations with OHRQoL [35].

Individuals with <20 teeth reported speech limitations. Phonetics was a concern when certain sounds were most affected or they had to cover their mouth due to missing teeth while speaking, thereby further worsening speech. Not many studies have looked at each item analysis within GOHAI scale; however, one study points out to lower GOHAI score with dentures inhibiting speech one month after insertion [36].

In OHIP-14, total inability to function was found to be significantly associated with fewer teeth and edentulousness. Patients with <20 teeth also reported problems with speech (Table 2).

OHIP-14 takes into consideration one year of time reference and places greater emphasis on more severe and less common psychosocial outcomes [11, 37]. In addition, OHIP can identify groups that prioritize their own treatment needs, oral health, and outcomes of dental care that increase QoL [14]. In our study, patients with <20 teeth and no teeth at all reported their inability to carry out daily activities. Either these HD patients prioritized their treatment needs or the severity of dental problems escalated to a point where it interfered with daily activities. Moreover, when recalling oral problems in the last year, people tend to forget minor discomfort and highlight more severe long-term outcomes.

Inability to function was also found significantly associated with race. While our study grouped diverse ethnicities such as Haitians into African Americans (AA), one of the limitations of the study, treatment needs were more evident among Black adults. They had a higher proportion (21.4%) of edentulousness compared to Whites (15.6%). Filled teeth index was lower for Black and Hispanics as compared to Whites showing disparities in access to dental care. Studies assessing OHRQoL found AA adults to report lower OHIP-14 scores [38–40].

There were eight significant associations of individual GOHAI-12 items with gender, age, years on dialysis, number of teeth, and three significant associations with number of teeth and race within the OHIP-14 scale. Within GOHAI-12, more than one-third of the participants reported two oral functional limitations, one pain/discomfort issue, and one psychosocial issue. In OHIP-14, more than one-third of the respondents reported one functional limitation, two pain/discomfort issues, and two psychosocial impacts. Lastly, fewer participants (12.9%) had perfect scores in GOHAI-12 than 20% in OHIP-14 suggesting GOHAI-12 to be more successful in detecting the impacts of oral disorders in this population. Therefore, the study found GOHAI-12 to be more effective in describing OHRQoL, which differed from the previous studies that found OHIP-14 to describe OHRQoL better [20, 41].

Most of the clinical findings did not compare with the subjective measures of oral health in the sample. Patients downplayed the impacts oral health had on their QoL despite clinical assessments that indicated poor oral health. Almost 87% of the participants were in need of oral prophylaxis, and higher than the national average (19% versus 17.6%) of adults >65 years were edentate in our sample. Evident through these assessments is a need for dental care, which is expensive and not easily available to HD patients, most of whom are Medicare beneficiaries. As previously proposed, there should be availability of dental package offered as a premium-financed voluntary insurance option under Medicare in addition to cooperation between nephrologists and dentists in treating systemic conditions and promoting oral health among HD patients [42, 43].

One of the limitations of the study was the small sample size, thus limiting our ability to detect significant difference between groups. Convenience sample might further limit generalizations to other populations. Intrarater and Interrater reliabilities were not determined, given the nature of the setting in which data acquisition took place and to not place significant burden on HD patients. Some discrepancies in the responses for similar questions in GOHAI-12 and OHIP-14 indicated HD patients who answered the surveys wanted to be seen as agreeable incorporating some response bias in the results.

## 5. Conclusion

To our knowledge, this is the first study to look at OHRQoL among HD patients in southwest Florida and finds GOHAI-12 to be more effective in assessing OHRQoL. HD patients, especially females, minorities, and those with fewer than 20 teeth reported trouble biting/chewing certain foods and discomfort in eating, were worried, were self-conscious, had speech-related problems, or were unable to function. However, most of the reported oral-health issues were not perceived to affect QoL to a large extent as their general health required more attention, and slow, gradual oral-health changes resulted in acceptance and adaptation of oral-health dysfunction and discomfort. The study highlights poor dental health among ESRD patients and the mismatch between clinical findings and subjective experiences. Further research on HD patients using QHRQoL scales and clinical findings is warranted to corroborate our findings.

## Figures and Tables

**Table 1:** Association of individual GOHAI-12 items with demographic and clinical characteristics of study participants.

Variable	*N*	GOHAI—physical				GOHAI—psychosocial					GOHAI—pain/discomfort		
		Trouble chewing	Comfortable to swallow	Prevented from speaking	Limit food	Pleased with looks	Worried	Self-conscious	Uncomfortable eating in front of people	Limit contact	Eat w/o discomfort	Use medication	Sensitivity
Gender													
Male	41	5 (2, 5)	5 (5, 5)	5 (5, 5)	5 (3, 5)	3 (1, 5)	4 (3, 5)	5 (4, 5)	5 (5, 5)	5 (5, 5)	5 (2, 5)	5 (3, 5)	5 (3, 5)
Female	27	3(1.75, 5) 0.62	5 (2, 5) 0.264	5 (3, 5) 0.243	4.5 (3, 5) **0.043**	4 (1.75, 5) 0.650	3 (1, 5) 0.221	5 (1.75, 5) 0.158	5 (3, 5) **0.045**	5 (2.75, 5) **0.046**	2.5 (1, 5) **0.011**	5 (2.75, 5) 0.419	5 (3, 5) 0.429

Race													
White	33	3 (2, 5)	5 (5, 5)	5 (5, 5)	5 (3, 5)	3.5 (2, 5)	5 (3, 5)	5 (5, 5)	5 (5, 5)	5 (2.75, 5)	4 (2, 5)	5 (3.25, 5)	4.5 (3, 5)
Black	28	5(2.75, 5)	5 (2.75, 5)	5 (2.75, 5)	5 (3, 5)	3 (.75, 5)	3 (1, 5)	5 (2, 5)	5 (2.75, 5)	5 (5, 5)	4 (1, 5)	5 (2.75, 5)	5 (3, 5)
Hispanic	08	5 (2, 5) 0.911	5 (5, 5) 0.425	5 (5, 5) 0.209	5 (3, 5) 0.743	4 (1, 5) 0.701	3 (3, 5) 0.134	5 (0, 5) 0.103	5 (0, 5) 0.178	5 (5, 5) 0.099	5 (2, 5) 0.868	5 (3, 5) 0.821	5 (3, 5) 0.886

Age													
≤65 years	30	3 (2.5, 5)	5 (4.5, 5)	5 (3, 5)	5 (2.5, 5)	3 (0.5, 5)	3 (1, 5)	5 (3, 5)	5 (3, 5)	5 (5, 5)	5 (3, 5)	5 (2, 5)	5 (2, 5)
>65 years	38	5 (2, 5) 0.936	5 (5, 5) 0.252	5 (5, 5) 0.122	5 (3, 5) 0.317	4 (2, 5) 0.415	5 (3, 5) **0.040**	5 (5, 5) **0.048**	5 (4.25, 5) 0.592	5 (5, 5) 0.397	5 (4.25, 5) 0.696	5 (4.25, 5) 0.078	5 (3, 5) 0.223

Teeth													
<20	27	3 (2, 5)	5 (4.5, 5)	5 (2.75, 5)	5 (2.75, 5)	3 (1.75, 5)	4.5(1.75, 5)	5 (3, 5)	5 (3, 5)	5 (4.5, 5)	5 (3, 5)	5 (3.75, 5)	4.5 (2, 5)
≥20	42	5 (2, 5) 0.395	5 (5, 5) 0.528	5 (5, 5) **0.016**	5 (3, 5) 0.612	3 (1, 5) 0.703	4 (2, 5) 0.889	5 (3, 5) 0.582	5 (4, 5) 0.218	5 (5, 5) 0.154	5 (4, 5) 0.551	5 (3, 5) 0.677	5 (3, 5) 0.643

Dental status													
Dentate	57	3 (2, 5)	5 (5, 5)	5 (4.5, 5)	5 (3, 5)	3 (1, 5)	4 (2, 5)	4 (2, 5)	5 (4, 5)	5 (5, 5)	4 (2, 5)	5 (3, 5)	5 (3, 5)
Edentate	12	4.5 (2, 5) 0.892	5 (3.25, 5) 0.328	5 (3, 5) 0.259	4 (2.25, 5) 0.497	4.5 (2.25, 5) 0.177	5 (1.5, 5) 0.512	5 (1.5, 5) 0.781	4 (2.25, 5) 0.122	5 (3.25, 5) 0.310	4.5 (2, 5) 0.570	5 (3.5, 5) 0.701	5 (4, 5) 0.586

Diabetes													
Yes	36	3 (2, 5)	5 (5, 5)	5 (5, 5)	5 (3, 5)	4 (2, 5)	5 (2, 5)	5 (5, 5)	5 (3, 5)	5 (5, 5)	4 (2, 5)	5 (4, 5)	5 (3, 5)
No	34	5 (2, 5) 0.552	5 (4.5, 5) 0.346	5 (3, 5) 0.583	5 (3, 5) 0.848	2.5 (.75, 5) 0.205	3 (2, 5) 0.295	5 (2, 5) 0.166	5 (3.75, 5) 0.925	5 (4.5, 5) 0.506	5 (1.75, 5) 0.469	5 (3, 5) 0.179	5 (2.75, 5) 0.697

CPI													
0	8	3 (1.5, 5)	5 (2.5, 5)	5 (3.5, 5)	5 (2.25, 5)	4.5(2.5, 5)	5 (3.5, 5)	5 (3.5, 5)	5 (5, 5)	5 (5, 5)	5 (5, 5)	5 (3.5, 5)	5 (3.5, 5)
1–4	50	5 (2, 5) 0.525	5 (5, 5) 0.160	5 (4.5, 5) 0.916	5 (3, 5) 0.917	3 (1, 5) 0.272	3 (2, 5) 0.186	5 (3.5, 5) 0.563	5 (3, 5) 0.323	5 (5, 5) 0.745	5 (3, 5) 0.756	5 (3, 5) 0.598	5 (3, 5) 0.431

Saliva pH													
Acidic	21	3 (1, 5)	5 (2.75, 5)	5 (2.75, 5)	5 (1.75, 5)	4.5(1.75, 5)	3 (1, 5)	5 (2.75, 5)	5 (1.75, 5)	5 (2.75, 5)	2 (1, 5)	5 (2.75, 5)	4 (3, 5)
Healthy	41	5 (3, 5) 0.143	5 (5, 5) 0.184	5 (5, 5) 0.769	5 (3, 5) 0.197	3 (1, 5) 0.176	4 (3, 5) 0.414	4 (3, 5) 0.451	5 (5, 5) 0.150	5 (5, 5) 0.108	4 (2, 5) 0.099	5 (4, 5) 0.603	5 (3, 5) 0.363

Years on dialysis													
≤3 years	41	5 (2, 5)	5 (5, 5)	5 (3, 5)	5 (3, 5)	3.5 (2, 5)	5 (3, 5)	5 (4, 5)	5 (3, 5)	5 (5, 5)	4 (1.25, 5)	5 (3.25, 5)	5 (3, 5)
>3 years	29	3 (2, 5) 0.394	5 (4.5, 5) 0.555	5 (4.5, 5) 0.631	4 (2.5, 5) 0.213	2 (1, 5) 0.233	3 (1.5, 5) **0.042**	5 (2.5, 5) 0.388	5 (3.5, 5) 0.527	5 (5, 5) 0.591	4 (2, 5) 0.875	5 (3, 5) 0.885	5 (3, 5) 0.692

For each GOHAI-12 item, the median and the IQR (in parentheses) for the categories are presented first, followed by the *p* value for comparison. The Mann–Whitney *U* test and Kruskal–Wallis test were used to compare two or more than two groups, respectively. *p* values that are in bold are statistically significant

**Table 2:** Association of individual OHIP items with demographic and clinical characteristics of study participants.

Variable	*N*	Functional limitation		Psychosocial impact								Pain/discomfort			
		Speech	Sense of taste	Self-conscious	Tension	Difficult to relax	Embarrassed	Irritable	Occupational	Unsatisfactory life	Unable to function	Painful aching	Uncomfortable eating	Unsatisfactory diet	Interrupt meals
Gender															
Male	41	5 (5, 5)	5 (3, 5)	5 (3, 5)	5 (5, 5)	5 (5, 5)	5 (3, 5)	5 (4, 5)	5 (5, 5)	5 (3, 5)	5 (5, 5)	5 (2, 5)	5 (2, 5)	5 (2, 5)	4 (4, 5)
Female	27	5 (5, 5) 0.754	5 (2, 5) 0.508	3 (3, 5) 0.090	5(4.75, 5) 0.886	5 (3, 5) 0.056	3.5(2.75, 5) 0.090	5 (3.75, 5) 0.948	5 (5, 5) 0.747	5 (3.75, 5) 0.754	5 (5, 5) 0.959	5 (3, 5) 0.876	5 (3, 5) 0.460	5 (3, 5) 0.812	5 (4, 5) 0.710

Race															
White	33	5 (5, 5)	5 (2.25, 5)	5 (3, 5)	5(4.25, 5)	5 (5, 5)	5 (3, 5) 03.5	5 (5, 5)	5 (5, 5)	5 (3.25, 5)	5 (5, 5)	5(3, 5)	4.5 (2, 5)	5 (2.25, 5)	5 (4, 5)
Black	28	5 (5, 5)	5 (2, 5)	3 (2.75, 5)	5 (5, 5)	5 (2.75, 5)	(2, 5)	5 (3, 5)	5 (3, 5)	5 (2.75, 5)	5 (4, 5)	5(3, 5)	5 (2, 5)	5 (2.75, 5)	5 (4.75, 5)
Hispanic	08	5 (2, 5) 0.263	5 (3, 5) 0.822	5 (1, 5) 0.396	5 (2, 5) 0.891	5 (5, 5) 0.107	5(2, 5) 0.143	5 (5, 5) 0.106	5 (5, 5) 0.190	5 (3, 5) 0.812	5 (5, 5) **0.018**	5 (2, 5) 0.865	3 (2, 5) 0.788	5 (5, 5) 0.500	5 (3, 5) 0.797

Age															
≤65 years	30	5 (5, 5)	5 (5, 2.5)	5 (3, 5)	5 (4, 5)	5 (3, 5)	5 (3, 5)	5 (3, 5)	5 (4.5, 5)	5 (3, 5)	5 (5, 5)	5 (2.5, 5)	4 (2, 5)	5 (5, 5)	5 (3.5, 5)
>65 years	38	5 (5, 5) 0.550	5 (5, 5) 0.915	5 (3, 5) 0.797	5 (5, 5) 0.624	5 (5, 5) 0.336	5 (3, 5) 0.312	5 (5, 5) 0.011	5 (5, 5) 0.464	5 (4, 5) 0.275	5 (5, 5) 0.666	5 (3, 5) 0.209	5 (2.25, 5) 0.586	5 (2.25, 5) 0.376	5 (5, 5) 0.484

Teeth															
<20	27	5 (3, 5)	5 (2, 5)	3.5 (3, 5)	5 (3, 5)	5(2.75, 5)	3.5(2.75, 5)	5 (3.75, 5)	5 (5, 5)	5 (2, 5)	5 (3.75, 5)	5 (3, 5)	5 (2, 5)	5 (2, 5)	5 (2.75, 5)
≥20	42	5 (5, 5) **0.024**	5 (2, 5) 0.755	5 (3, 5) 0.463	5 (5, 5) 0.059	5 (4, 5) 0.516	5 (3, 5).174	5 (4, 5) 0.911	5 (5, 5) 0.832	5 (3, 5) 0.404	5 (5, 5) **0.028**	5 (3, 5) 0.437	5 (2, 5) 0.779	5 (5, 5) 0.306	5 (5, 5) 0.058

Dental status															
Dentate	57	5 (5, 5)	5 (2, 5)	5 (3, 5)	5 (5, 5)	5 (4, 5)	5 (3, 5)	5 (4, 5)	5 (5, 5)	5 (3, 5)	5 (5, 5)	5 (3, 5)	5 (2, 5)	5 (3, 5)	5 (4.5, 5)
Edentate	12	5(3.5, 5) 0.398	5 (2.5, 5) 0.837	4 (2, 5) 0.401	5 (4, 5) 0.463	5 (3, 5) 0.519	4.5 (2.25, 5) 0.475	5 (3.5, 5) 0.984	5 (5, 5) 0.98	5 (3, 5) 0.907	5 (3, 5) **0.031**	5 (3.5, 5) 0.401	5 (2, 5) 0.830	5 (3.25, 5) 0.759	5 (3.25, 5) 0.531

Diabetes															
Yes	36	5 (5, 5)	5 (3, 5)	4 (3, 5)	5 (5, 5)	5 (5, 5)	5 (3, 5)	5 (5, 5)	5 (5, 5)	5 (4, 5)	5 (5, 5)	5 (3, 5)	5 (2, 5)	5 (3, 5)	5 (4, 5)
No	34	5 (5, 5) 0.926	5 (2, 5) 0.405	5(2.75, 5) 0.715	5 (4.5, 5) 0.809	5 (2.75, 5) 0.088	5 (2.75, 5) 0.920	5 (3, 5) 0.160	5 (4.5, 5) 0.200	5 (2.75, 5) 0.577	5 (4.75, 5) 0.176	4.5 (2, 5) 0.110	5 (2, 5) 0.878	5 (2, 5) 0.717	5 (3.75, 5) 0.613

CPI															
0	8	5(3.5, 5)	3.5(1.25, 5)	5 (3, 5)	5(2.25, 5)	5 (5, 5)	5 (2.25, 5)	5 (3.5, 5)	5 (5, 5)	5 (2.75, 5)	5 (5, 5)	5(2.25, 5)	5 (3.5, 5)	5 (2.75, 5)	5 (3.5, 5)
1–4	50	5 (5, 5) 0.357	5 (3, 5) 0.288	5 (3, 5) 0.554	5 (5, 5) 0.222	5 (3, 5) 0.413	5 (3, 5) 0.949	5 (4, 5) 0.930	5 (5, 5) 0.775	5 (3, 5) 0.134	5 (5, 5) 0.936	5 (3, 5) 0.970	4 (2, 5) 0.176	5 (3, 5) 0.899	5 (4, 5) 0.930

Saliva pH															
Acidic	21	5(4.75, 5)	5 (2, 5)	5(2.75, 5)	5(3.75, 5)	5 (3, 5)	5 (2.75, 5)	5 (3, 5)	5 (4.75, 5)	5 (2.75, 5)	5 (5, 5)	5 (3, 5)	4.5 (2, 5)	5 (2.75, 5)	5 (4, 5)
Healthy	41	5 (5, 5) 0.245	5 (2, 5) 0.918	5 (3, 5) 0.833	5 (5, 5) 0.405	5 (4, 5) 0.762	5 (3, 5) 0.913	5 (5, 5) 0.530	5 (5, 5) 0.727	5 (3, 5) 0.581	5 (5, 5) 0.990	5 (3, 5) 0.987	5 (2, 5) 0.590	5 (3, 5) 0.857	5 (4, 5) 0.599

Years on dialysis															
≤3 years	41	5 (5, 5)	5 (2.75, 5)	4 (3, 5)	5 (4.5, 5)	5 (4.75, 5)	5 (3, 5)	5 (3.75, 5)	5 (5, 5)	5 (3, 5)	5 (5, 5)	5 (3, 5)	5 (2, 5)	5 (2.75, 5)	5 (4.5, 5)
>3 years	29	5 (5, 5) 0.686	5 (2, 5) 0.797	5 (3, 5) 0.284	5 (5, 5) 0.590	5 (3, 5) 0.279	5 (2, 5) 0.735	5 (4, 5) 0.640	5 (5, 5) 0.843	5 (4, 5) 0.544	5(5, 5) 0.631	5 (3, 5) 0.771	4 (2, 5) 0.550	5 (3, 5) 0.747	5 (4, 5) 0.738

For each OHIP-14 item, the median and the IQR (in parentheses) for the categories are presented first, followed by the *p* value for comparison. Mann–Whitney *U* test and Kruskal–Wallis test were used to compare two or more than two groups, respectively. *p* values that are in bold are statistically significant.

**Table 3:** Mean and standard deviations of decayed, missing, filled teeth index, CPI, and saliva PH across demographic and clinical characteristics of study participants and number and percentage (in parentheses) of edentate patients followed by the total number (*n*) of participants in that category.

Variable	Decayed (*T*)	Missing (*T*)	Filled (*T*)	CPI	Saliva pH	Edentulousness
Gender						
Male	1.7 (3.4)	12.0 (11.1)	3.6 (4.8)	1.8 (1.0)	6.9 (1.1)	9 (22.0) (41)
Female	1.4 (1.9)	9.5 (9.6)	1.9 (3.8)	1.7 (1.0)	6.8 (0.9)	3 (11.5) (26)
Race/ethnicity						
White	2.2 (3.8)	12.1 (10.0)	4.8 (5.3)	1.9 (1.2)	7.0 (1.2)	5 (15.6) (32)
Black	0.8 (1.5)	11.0 (11.5)	1.0 (2.2)	2.0 (0.8)	7.0 (0.8)	6 (21.4) (28)
Hispanic	1.6 (1.9)	7.0 (8.9)	2.8 (4.0)	1.6 (1.1)	6.5 (1.0)	0 (0) (8)
Age groups						
≤65 years	1.6 (3.0)	9.1 (10.6)	2.1 (3.1)	2.0 (1.1)	6.8 (0.9)	4 (13.3) (30)
≥66 years	1.5 (2.8)	12.6 (10.3)	3.8 (5.3)	1.7 (1.0)	7.0 (1.1)	7 (19.0) (37)
Diabetes						
Yes	1.5 (2.3)	12.4 (11.2)	3.1 (4.5)	1.9 (1.0)	6.8 (1.1)	7 (19.4) (36)
No	1.6 (3.4)	10.2 (10.1)	2.9 (4.5)	1.9 (1.0)	7.0 (1.0)	5 (15.2) (33)
Years on dialysis						
≤3 years	1.5 (2.8)	12.8 (11.1)	2.8 (4.6)	1.8 (1.0)	7.0 (1.0)	8 (20) (40)
>3 years	1.7 (3.0)	9.3 (9.9)	3.2 (4.4)	2.0 (1.0)	6.8 (1.1)	4 (13.8) (29)
Overall	1.5 (2.9)	11.3 (10.7)	3.0 (4.5)	1.9 (1.0)	6.9 (1.0)	12 (17.1) (70)

## Data Availability

Readers can access the data upon sending a request to the first author.
